# In-Hospital Mortality following Proximal Femur Fractures in Elderly Population

**DOI:** 10.1055/s-0039-1692995

**Published:** 2019-07-16

**Authors:** Ganesan G. Ram, Praveen Govardhan

**Affiliations:** 1 Department of Orthopaedics, Sri Ramachandra Medical College, Porur, Chennai, India; 2 Department of Orthopaedics, Vasanthi Orthopaedic Hospital, Arumbakkam, Chennai, India

**Keywords:** mortality, proximal femur, Parker's mobility score, replacement surgeries, elderly

## Abstract

**Context** In India, hip fracture crude incidence above the age of 50 years was 129 per 100,000.

**Aims** The aim of this study is to analyze the in-hospital mortality following proximal femur fractures in elderly Indian population.

**Methods and Material** The study was done in Sri Ramachandra Medical Center, Chennai, India. Patient's records were retrospectively evaluated for a period of 3 years from January 1, 2015 to January 1, 2018. The inclusion criteria were patients both male and female aged more than 65 years admitted with the diagnosis of neck of femur or intertrochanteric or subtrochanteric fractures. The exclusion criteria were patients having any associated fracture or previous hip fracture history or diagnosed primary or secondary malignancies. To evaluate any surgical delay two groups were formed. After eliminating cases based on exclusion criteria, we had 270 patients for evaluation.

**Statistical Analysis Used** The collected data were analyzed with IBM.SPSS statistics software 23.0 Version. To describe about the data descriptive statistics frequency analysis, percentage analysis were used for categorical variables and the mean and standard deviation (SD) were used for continuous variables. To find the significant difference between the bivariate samples, Student's *t* -test and analysis of variance (ANOVA) were used. The *p* -value of 0.05 is considered as significant level.

**Results** We had a total of 24 mortalities with 15 males and 9 females. The in-hospital mortality of patients who underwent replacement surgeries for proximal femur fractures was 14 in our study. Sixteen of the in-hospital mortality patients had low Parker's mobility score. Twenty patients had mortality when surgery was delayed more than 48 hours.

**Conclusions** In-hospital mortality in elderly patients having proximal femur fracture increases significantly if the patient was having low-preoperative mobility status, if surgery was delayed more than 48 hours, and if patient undergoes replacement surgeries.

In-hospital mortality in proximal femur fracture increases on preoperative mobility status delay in surgery and in prosthetic replacements.

A Proximal femur fractures is a broad term involving the neck of femur, pertrochantric, and subtrochanteric region fractures. 1 The 90 to 95% of proximal femur fractures are femoral neck fractures and pertrochanteric fractures and remaining 5 to 10% are subtrochanteric fractures. 2 3 Out of all fractures 14% fractures are proximal femur fractures and it accounts for nearly 72% of total value for the treatment of fractures. Lifetime risk of hip fracture was 23.3% for men and 11.2% for women. 4 The increase in incidence of hip fractures with increasing the age is a result of an age-related decrease of bone mass in the proximal femur, as well as of the age-related increase in the incidence of falls. In patients over 65 years of age, fractures of the hip were associated with approximately double the mortality of the general population. For hip fractures Standardized Mortality Rate is 2.0 for women and 3.0 for men. 5 In India, Hip fracture crude incidence above the age of 50 years was 129 per 100,000. 6 There are various studies pertaining to the early mortality of proximal femur fractures all over the world and none from subcontinent. The aim of this study was to analyze the in-hospital mortality following proximal femur fracture in elderly Indian population.

## Subjects and Methods

The study was done in Sri Ramachandra Medical Centre, a tertiary care referral center in Chennai, South India. Patients' records were retrospectively evaluated for a period of 3 years from January 1, 2015 to January 1, 2018. The hospital database has all the necessary documents like diagnosis, comorbidities, surgical intervention, deep vein thrombosis prophylaxis, time in hospital from admission to discharge, or death. This database was linked to individual records of mortality. The inclusion criteria were patients both male and female aged above 65 years admitted with the diagnosis of neck of femur or intertrochanteric or subtrochanteric fractures. Ageing, an inevitable process is commonly measured by chronological age and, as a convention, a person aged 65 years or more is often referred to as “elderly.” 7 8 . The modality of fracture fixation was either bipolar hemiarthroplasty or total hip arthroplasty or dynamic hip screw fixation or proximal femur plate or dynamic condylar screw or proximal femur nail. The patients who died during preoperative hospital stay were also included. The exclusion criteria were patients having any associated fracture or previous hip fracture history or diagnosed primary or secondary malignancies. Approval obtained from Sri Ramachandra Medical College institutional ethics committee.

Each patient's number of comorbidities was taken from the patient's records of the previous 2 years. Only the comorbidities like diabetes mellitus, obesity, dementia, Parkinson's disease, hypertension, cerebrovascular diseases, vascular diseases, chronic nephropathy, chronic diseases, rheumatoid arthritis, and osteoporosis were considered. For all the patients included the Charlson's comorbidity index calculated and those having more than 5 were excluded. 9 To investigate whether there was a difference in mortality between the most common interventions, four groups were included in the analysis. They were conservative group (preoperative death), intramedullary group, extramedullary group, and replacement group. In intramedullary group, patients who underwent proximal femur nail, cervicotrochantric nails were included. In extramedullary group, proximal femur plating, dynamic condylar screw, and dynamic hip screw were included. In replacement group, the hemiarthroplasty, bipolar and total hip arthroplasty patients were included. All the patients, who underwent surgery, were mobilized from 2nd postoperative day either partial or full weight bearing.

The preinjury mobility statuses of all patients were calculated using Parker's mobility score. Patients were divided as low, medium, and high. The Parker's mobility score is an assessment tool that ranks pre fracture mobility on a scale of 0 to 9. A person with a score of 9 is independent in mobility at home and in the community, whereas someone with a score of 0 is completely dependent for ambulation. 10 To evaluate any surgical delay two groups were formed. First group contains patients who were operated within 48 hours of injury, while the second group had patients operated after 48 hours of injury. After double verification of data by two separate data analyzers', we had 283 patients who fulfilled the inclusion criteria. After eliminating cases based on exclusion criteria we had 270 patients for evaluation. Out of which 151 were females and 119 were males. We used one-way analysis of variance (ANOVA) *F* -test for statistical analysis of in hospital mortality with preinjury status of the patients and Student's independent *t* -test for statistical correlation of mortality with various methods of treatment given, sex of the patients, age of the patients, and surgical timing.

## Results

We had a total of 24 mortalities with 15 males and 9 females. The in-hospital mortality following proximal femur fracture in elderly population was 10.03%. Twenty patients had mortality when surgery was done for more than 48 hours ( Table 1 ). Table 2 highlights that 14 patients had in-hospital mortality when they underwent replacement surgeries for proximal femur fracture. We had 14 patients aged above 70 years died in hospital during the study period. Sixteen of the in-hospital mortality patients had low Parker's mobility score ( Table 3 ).

**Table 1:** Mortality based on surgical delay

	Replacement group		Intramedullary group		Extramedullary group		Conservative group	
	Surgery within 48 h	Surgery after 48 h	Surgery within 48 h	Surgery after 48 h	Surgery within 48 h	Surgery after 48 h	Death within 48 h	Death after 48 h
No. of mortalities (24)	2	12	1	3	1	1	–	4
Total no of patients	135		75		56		4	

**Table 2:** Correlation of mortality and treatment methods

Method of treatment	Number of mortality	Mean	Standard deviation	Student's independent *t* -test
Replacement	14	23.42	25.60	*t* = 2.12; *p* = 0.05 significant
Fixation/conservative	10	5.90	4.97	

**Table 3:** Mortality based on preinjury status of patients

Parker's score	Number of mortality	Mean	Standard deviation	One-way ANOVA *F* -test
Low (0–4)	16	21.62	24.54	*F* = 3.91; *p* = 0.02 significant
Medium (5–8)	6	5.16	3.06	
High (9)	2	5.00	1.41	
Total	24	16.1250	21.40563	

Abbreviation: ANOVA, analysis of variance.

## Discussion

Hip fractures in elderly increases the morbidity and mortality considerably. 11 12 In-hospital mortality rates ranges between 1 and 10%. 12 13 14 15 16 Mortality in proximal femur fractures was considered high in male sex and in elderly population. We had 14 patients aged more than 70 years who had in-hospital mortality, which is not statistically significant ( Table 4 ), which disproves the common belief that as age increases mortality increases. Table 5 proves that there is no correlation between genders when considering in-hospital mortality in proximal femur fractures. Surgical treatment has been established as the gold standard; however, the surgical option remains a dilemma as none of the existing osteosynthesis devices could prove its superiority in previous studies. 17 18 Parker and Gurusamy in a systematic review of this subject that included 19 trials, internal fixation was found to result in lower morbidity in several categories, including blood loss and risk of deep wound infection. 19 However, patients treated with arthroplasty had significantly lower reoperation rates. No differences were identified in mortality or regaining previous residential status. As per Sathiyakumar et al, retrospective analysis of 9,640 patients undergoing operative repair of a hip fracture open reduction and internal fixation of femoral neck fractures was associated with the highest percentage of total adverse events and major adverse events (primarily death). Whereas hemiarthroplasty was associated with a higher percentage of minor adverse events (e.g., urinary tract infection). 20 The current study on hospital mortality when a patient underwent replacement was 14, which is statistically significant when compared to the other methods of treatment as evident from Table 2 .

**Table 4:** Correlation of mortality and age of patients

Age	Number of mortality	Mean	Standard deviation	Student's independent *t* -test
> 70 y	14	20.00	26.68	*t* = 1.05; *p* = 0.30 not significant
65–70 y	10	10.70	9.29	

**Table 5:** Correlation of mortality and sex of patients

Sex	Number of mortality	Mean	Standard deviation	Student's independent *t* -test
Male	15	14.93	17.28	*t* = 0.34; *p* = 0.73 not significant
Female	9	18.11	28.05	

The patients with lower Parker's mobility score had a statistically significant mortality rate compared to the patients who had higher score ( Table 3 ). The recommended guidelines for the management of proximal femur fracture were within 2 days. 10 There was literature support for and against the early management of proximal femur fractures. Some studies have reported no differences in outcomes between early and late management, 21 while others reported that mortality increases only if surgery is delayed beyond the 4th day. 22 23 24 25 The present study from Table 6 clearly states that the in-hospital mortality rate increases significantly when surgery was delayed more than 48 hours.

**Table 6:** Correlation of mortality with timing of surgery

Surgery time	Number of mortality	Mean	Standard deviation	Student's independent *t* -test
> 48 h	20	12.75	15.30	*t* = 1.81; *p* = 0.08 significant
< 48 h	4	33.00	39.68	

## Limitations

The limitations of the study were retrospective and the study was done in a single center and involving many surgeons with varied experience which may alter the prognosis. The term “elderly population” was very tricky as it may vary based on different ethnicity. Moreover in the study, we didn't analyze the mortalities in different types of replacement surgeries like hemiarthroplasty, bipolar arthroplasty and total hip replacement that may affect the outcome. The hospital database from which the patients' details were obtained was not created exclusively for epidemiological analyses.

## Conclusion

Age and sex of the patients do not affect the in-hospital mortality in elderly. In-hospital mortality in elderly patients having proximal femur fracture increases significantly if the patient was having low-preoperative mobility status, if surgery was delayed more than 48 hours and if patient undergoes replacement surgeries.
